# Exploring crystal structure–physical property relationships with pressure

**DOI:** 10.1107/S205225252400602X

**Published:** 2024-06-27

**Authors:** David R. Allan

**Affiliations:** a Diamond Light Source Ltd Diamond House Chilton OxfordshireOX11 0DE United Kingdom

**Keywords:** intermolecular interactions, polymorphism, crystallization and crystal growth, properties of solids, hydrogen bonding, density functional theory, lattice energies, molecular crystals, structure prediction

## Abstract

From its conception, X-ray crystallography has provided a unique understanding of the structure, bonding and electronic state of materials, which, in turn, unlocks a means of examining the properties and function of crystalline systems. Using state-of-the-art single-crystal X-ray diffraction, along with UV–Vis spectroscopy and DFT calculations, Zwolenik *et al.* [(2024). *IUCrJ*, **11**, 519–527] have provided a comprehensive study of the structure–optical property relationship of 1,3-di­acetyl­pyrene with methodologies that are increasingly accessible to non-specialist laboratories.

In this issue of **IUCrJ**, Zwolenik *et al.* (2024 ▸) present a study of the evolution of the crystal structure and spectroscopic properties of a previously unobserved polymorph of 1,3-di­acetyl­pyrene under both variable temperature and pressure. This material is an example of a molecular conjugated system; these systems are of significant interest for their structure–property relationships. The new β-form polymorph is structurally distinct from the α-form [also reported by the authors (Tchoń & Makal, 2021 ▸)], as the molecules are arranged in π-stacked layers, which leads to pronounced photoluminescence. The authors studied the systems piezochromism and piezo­fluoro­chromism and their relationship with the variation in interplanar π-stacking distances using single-crystal X-ray diffraction, UV–Vis fluorescence spectroscopy and DFT calculations. Their results show that the optical properties relate directly to the variation of the interplanar distances within the π-stacking.

The properties of a material are intrinsically linked to its structure at an atomic level, and it was with the development of X-ray crystallography that these fundamental relationships could begin to be unpicked with unprecedented detail. The foundational X-ray diffraction studies were performed by William Lawrence Bragg and William Henry Bragg who, in one of their seminal papers of 1913, determined the structures of zincblende along with the simple alkali halides, sodium chloride and potassium chloride (Bragg, 1913 ▸). For what would now be considered extremely well-known and comparatively simple structures, their discovery proved revolutionary and controversial, with contemporary chemists not readily accepting them. In sodium chloride, for example, no individual molecules were observed, and this profoundly contradicted what was expected from molecular theory at the time, where the precise integer stoichiometries were considered to be a consequence of the valency of atoms in molecules (Authier, 2013 ▸). Even after fourteen years, with the X-ray diffraction technique becoming more established, the leading chemist, H.E. Armstrong, still found Bragg’s structure for sodium chloride ‘more than repugnant to the common sense, not chemical cricket’ and ‘chemistry is neither chess nor geometry, whatever X-ray physics may be’ (Armstrong, 1927 ▸). However, the profoundly definitive nature of X-ray crystallography was demonstrated unequivocally in 1929 with the determination of the molecular structure of benzene by Kathleen Lonsdale (Lonsdale, 1929 ▸). The shape of the molecule had been the subject of significant conjecture since its discovery by Michael Faraday in 1825 (Julian, 1981 ▸) with August Kekulé, who established the theory of chemical structure through valence (Kekulé, 1857 ▸), providing a preliminary model of the benzene structure in 1865 (Kekulé, 1865 ▸). The structure was the subject of speculation over the next few decades with the X-ray crystallography studies of Lonsdale, finally, providing a definitive resolution to the conjecture – offering new insights into the nature of the bonding within the benzene molecule and the electron-sharing nature of this archetypal conjugated system.

From its inception, therefore, X-ray crystallography studies not only provided a means of observing atomic positions within crystalline solids, but they also gave a unique insight into the nature of bonds, providing an unparalleled means of examining the properties and potential function of materials. The study of the correlation between the properties of crystalline materials and their structure has undergone considerable growth during the intervening period and encompasses a variety of physical effects, such as electrical conductivity, magnetism, catalysis and optical properties (Konar & Vittal, 2019 ▸). The use of the thermodynamic variables of temperature and pressure can strongly influence these physical properties, which can, in turn, be mapped to the accompanying variation of the structure. As the variable of pressure provides the most direct means of altering the chemical bonding and structure of a material, and it can be varied in the laboratory by several orders of magnitude (in comparison to temperature) from ambient to megabars, its application can, consequently, have a profound effect on the properties of a system (Tse, 2020 ▸). A prominent example is the observation of superconductivity in dense metallic hydrides, with very high critical temperatures (*T*_c_) reported in compressed hydrogen sulfide [200 K, Drozdov *et al.* (2015 ▸)] and lanthanum hydride [260 K, Somayazulu *et al.* (2019 ▸)].

The extreme pressures required are often generated through the use of diamond-anvil cells that allow the sample to be probed with X-rays, for crystallographic studies and, as diamond is also sufficiently transparent to electromagnetic radiation across a wide energy range, the cells can also be used for both IR and UV–Vis spectroscopy. The diamond-anvil cell does impose restrictions on the level of data that can be collected, however, and for IR spectroscopy type-II diamonds (which are essentially free of nitro­gen impurities) are required as they have low luminescence and are transparent to IR radiation (Mao, 2007 ▸). For single-crystal X-ray diffraction, the volume of accessible reciprocal space is restricted by the opening angle of the X-ray apertures and limits the completeness of a data set – particularly for low-symmetry systems. To improve completeness, two or more crystals can be loaded with sufficiently random relative orientations so that the portion of reciprocal space from each sample can be summed into a more complete set of data or, the sample can be mounted in the cell with an orientation that maximizes the potential data completeness for the crystal symmetry (Tchoń & Makal, 2021 ▸). The latter approach was adopted by Zwolenik, Tchoń and Makal who further improved both the completeness and resolution using synchrotron radiation, which can access X-ray wavelengths shorter than can be achieved on a lab source (Zwolenik *et al.*, 2024 ▸). They also adopted Hirshfeld Atom Refinement (HAR), which moves away from the more generally adopted spherical atom approximation and uses repeated *ab initio* molecular density calculations to successively determine the aspherical atomic scattering factors between cycles of least-squares structural refinement, with the calculated densities and refinements iteratively reaching convergence (Capelli *et al.*, 2014 ▸). This quantum crystallography approach (Grabowsky *et al.*, 2017 ▸) can provide dramatically improved refinement of atomic positions and ADPs for light atoms, particularly hydrogen atoms, and, in the current work, gave demonstrably improved structural models for the 1,3-di­acetyl­pyrene molecules.

To provide a deeper insight into the observed structural changes and the variation in the optical properties, Zwolenik, Tchoń and Makal utilized DFT calculations (Becke, 2014 ▸) to determine how the compression of the π-stacks affected the energy gap. They found that the accompanying DFT simulations accurately calculated the piezochromism associated with the π-stacking, which gave a 50 nm red-shift of the emission maximum in the high-pressure luminescence spectra from ambient pressure to 4 GPa. The work, therefore, provides a complete overview of the structure–optical property changes of the β-form of 1,3-di­acetyl­pyrene with methodologies that are, encouragingly, becoming increasingly accessible to non-specialist laboratories.
